# Artificially Created Nucleic Acids and Peptides/Proteins in Chemical Biology

**DOI:** 10.1155/2013/219263

**Published:** 2013-01-15

**Authors:** Masayasu Kuwahara, Yingfu Li, Eriks Rozners, Hiroshi Murakami

**Affiliations:** ^1^Graduate School of Engineering, Gunma University, Kiryu 376-8515, Japan; ^2^Departments of Biochemistry and Biomedical Sciences and Chemistry and Chemical Biology, Michael G. DeGroote Institute for Infectious Disease Research, McMaster University, Hamilton, ON, Canada L8S 4K1; ^3^Department of Chemistry, Binghamton University, State University of New York, Binghamton, NY 13902, USA; ^4^Graduate School of Arts and Sciences, The University of Tokyo, Komaba, Meguro-ku, Tokyo 153-8902, Japan

Nucleic acids—DNA and RNA—have been chosen by Mother Nature as the key players for orchestrating the preservation, transfer, and expression of genetic information in all the biological systems on Earth. RNA has also been enlisted to carry out other important cellular functions, such as catalysis and molecular recognition. Within the hands of scientists, the function of nucleic acids has been significantly expanded beyond what is known in nature, and as a result, we are now in the possession of a large array of synthetic, nucleic acid-based catalysts (ribozymes and DNAzymes) and receptors (DNA and RNA aptamers). DNA as a genetic material itself has also been subjected to various chemical modifications in efforts to derive significantly altered or even completely new genetic systems. These systems can be used to create novel peptides and proteins that offer enhanced activities or even completely new properties over their natural protein counterparts. Furthermore, many artificially engineered nucleic acids and proteins have found useful applications as biosensors, diagnostic agents, and therapeutic drugs. This special issue is assembled to reflect recent progress in the important research arena of artificially engineered nucleic acids and proteins.

This issue comprises 10 reviews and 7 research articles that can be grouped into three sections. The first section deals mainly with research on xeno-nucleic acids (XNAs)—nonnatural nucleic acid analogs with significantly altered sugar and/or phosphate backbones. D.-A. Catana et al. provide a review on the use of dinucleotides of dioxaphosphorinane-constrained nucleic acids (CNAs) to tune nucleic acid structures. This is followed by a review by E. Rozners on recent advances in chemical modifications of peptide nucleic acids (PNAs). G. Upert et al. then present a research article on designing cyclic and hairpin PNAs as inhibitors for HIV replication. Z. Wang et al. also present a research article where PNA probes were utilized for live cell imaging of mRNA expression. In their research article, T. Yamamoto et al. examine the gene-silencing effect of bridged/locked nucleic acids (BNA/LNAs). This section is closed out with a research article by S. Saxena et al. in which the molecular crowding effect on the structure and function of RecG (a helicase) was examined.

The second section includes four reviews and three research articles discussing the creation of novel peptides, proteins, transfer RNAs, and peptide mimicries using various selection or screening methods. K. Fukunaga and M. Taki provide a review on the phage display technique with a particular focus on tips for conducting successful phage display experiments. Within the same topic, T. Matsubara reviews the use of phage display for the creation of carbohydrate-mimetic peptides. These are followed by a research article by T. Sumida et al. exploiting the mRNA display technique for the selection of anti-p53 Fab fragments. There are two papers concerning the *in vitro* compartmentalization (IVC) technique, which offers an excellent way to link a genotype to a phenotype in a physically confined environment: the first is a review article by T. Nishikawa et al. on evolving proteins using IVC and the second is a research article by A. Ogawa et al. where IVC was used to select functional transfer RNAs. T. Kawakami and H. Murakami discuss, in their review article, potential applications of a translation system with a reprogrammed genetic code to prepare a peptide mimetic library. Finally, J. K. Pokorski and D. H. Appella present an on-bead screening approach to create peptide mimicries.

The last section of the issue consists of four review papers on the selection and application of functional nucleic acids. M. McKeague and M. C. DeRosa survey DNA and RNA aptamers derived by SELEX (systematic evolution of ligands via exponential enrichment) for small molecule binding, along with the compilation of nearly 40 improved methodologies of SELEX. K. Tram et al. review the application of fluorescently dressed RNA-cleaving DNAzymes for biosensing. Y. M. Chang et al. discuss the cell-SELEX technique for biomarker discovery. In addition, Y. Kasahara and M. Kuwahara provide a review on SELEX experiments exploring chemically modified nucleic acid libraries.

The ultimate goal in this research arena is to generate scientific knowledge and produce new technologies that will enable the engineering of novel or improved molecular systems to control biological activities at will. Along the way, researchers may help shine light on one of the biggest mysteries surrounding the origin and evolution of life on Earth—the question of why Mother Nature has selected 4 nucleotides to construct nucleic acids and 20 amino acids to construct proteins. We hope this special issue can further spur research efforts in this area.

